# Synergistic Effects of Organosilicon and Cu(OH)_2_ in Controlling Sugarcane Leaf Scald Disease

**DOI:** 10.3390/ijms232113532

**Published:** 2022-11-04

**Authors:** Ming-Yang Zhang, Ding-Kai Hong, Yao-Hui Chen, San-Ji Gao, Hua-Ying Fu, Hua-Kun Zheng, Yong Fang, Jin-Da Wang

**Affiliations:** 1 National Engineering Research Center of Sugarcane, Fujian Agricultural and Forestry University, Fuzhou 350002, China; 2 National Engineering Research Center of Juncao, Fujian Agricultural and Forestry University, Fuzhou 350002, China; 3 Putian Agricultural Science Research Institute, Fujian Academy of Agriculture Science, Putian 351106, China; 4 Hunan Agricultural Biotechnology Research Institute, Hunan Academy of Agriculture Science, Changsha 410125, China

**Keywords:** sugarcane disease, *Xanthomonas albilineans*, bactericide, silicon, disease resistance

## Abstract

Sugarcane leaf scald is a systemic disease caused by *Xanthomonas albilineans* that limits sugarcane yield and quality. Previous research has shown that exogenous application of copper hydroxide to plants is effective in controlling this disease. However, long-term bactericide use causes serious “3R” problems: resistance, resurgence, and residue. It is therefore urgent to discover new methods for the improvement of bactericide efficiency and efficacy. In the present study, disease index values for leaf scald were measured in sugarcane seedlings over time to determine the effects of different concentrations of copper hydroxide, types of silicon additive, and treatment timing after inoculation with *X. albilineans* on controlling sugarcane leaf scald disease. Our results show copper hydroxide mixed with organosilicon additive could improve the bactericide efficiency and efficacy and reduce the growth of pathogenic bacteria, even at a reduced concentration in both laboratory and field conditions. This study provides an important practical model for controlling sugarcane leaf scald disease by reducing the concentration of bactericide and increasing its efficacy in sugarcane fields.

## 1. Introduction

Sugarcane (*Saccharum* spp. hybrids) is the most important sugar crop in the world, and ~60% of the total sugar [1] in the world and ~90% of the total sugar in China are produced from sugarcane [2]. Because sugarcane reproduces asexually, successive years of lodging lead to repeated infestations and accumulation of multiple pathogens in plants. This accumulation triggers sugarcane diseases, resulting in reduced yield and sugar content, which have huge negative impacts on the development of the sugar industry in China. Sugarcane leaf scald disease is a systemic infection caused by *Xanthomonas albilineans* (*Xa*) [3]. This pathogenic bacterial species parasitizes the vascular bundle of sugarcane, causing impaired chlorophyll differentiation in leaves and producing leaf scald symptoms. At later stages, leaf scalds merge to cause symptoms such as leaf wilting and plant death. Since the first detection of *Xa* in Hainan province in 1983, this disease has become widespread among all sugarcane production areas in China, greatly threatening local sugarcane production [4,5].

Currently, the most effective methods of controlling sugarcane leaf scald disease rely on the selection of resistant varieties and seedling disinfection or detoxification [6]. However, outbreaks of this disease still cause a great deal of damage. Management strategies therefore increasingly focus on plant treatment with chemical bactericides such as 14% cupric tetramminosulfate or 6% kasugamycin wettable powder [7,8]. Even when these bactericides are heavily applied, the control effects remain relatively low. There is thus an urgent need to either identify alternatives to chemical fungicides or to reduce the volume of its application while improving effectiveness.

Organic silicon is widely used as a fungicide additive. It can improve the utilization rate and efficacy of fungicide agents by increasing retention of fungicide solution on the plant surface, reducing surface tension of the fungicide solution, and improving resistance of the fungicide solution to rainwater washing. Many studies have shown enhanced fungicide efficacy with the addition of silicons to fungicide solutions. In a field efficacy trial of fungicides against the powdery mildew of grape, Jjiang et al. showed that the “jiexiaoli” synergist had a significant synergistic effect on 29% isopyrazam·azoxystrobin 29% SC, and the efficacy of treatments with this synergist was better than that of single-agent treatments when reduced by 30% [9]. Ren et al. Showed that the average control effect of Prochloraz and Prochloraz plus organic silicone against Sclerotinia sclerotiorum was 74.0% and 80.0% respectively, suggesting that the addition of organic silicone had a certain synergism on the control of Sclerotinia sclerotiorum by Prochloraz [10]. Silicon has shown similarly positive synergistic effects with other agents, such as carbendazim, triadimefon [11], chlorpyrifos 30% EW, and ZJ0712 SC [12], in controlling the corresponding pests and diseases and in effectively reducing the amount of each agent required.

Previous research by our group showed that copper hydroxide was effective in suppressing the occurrence of sugarcane leaf scald disease. In this study, we determined the minimum required concentration of copper hydroxide and the effects of silicon additives under growth chamber conditions. The results were then validated with a field experiment. Our results provide both an effective method for controlling sugarcane leaf scald and an example for improved bactericide efficiency through inclusion of silicons in bactericide preparations. These findings contribute to the key goals of reducing chemical bactericide application and increasing the efficiency of those that are applied.

## 2. Results

### 2.1. The Optimum Concentration of Copper Hydroxide for Controlling Sugarcane Leaf Scald Disease

**Figure 1:**
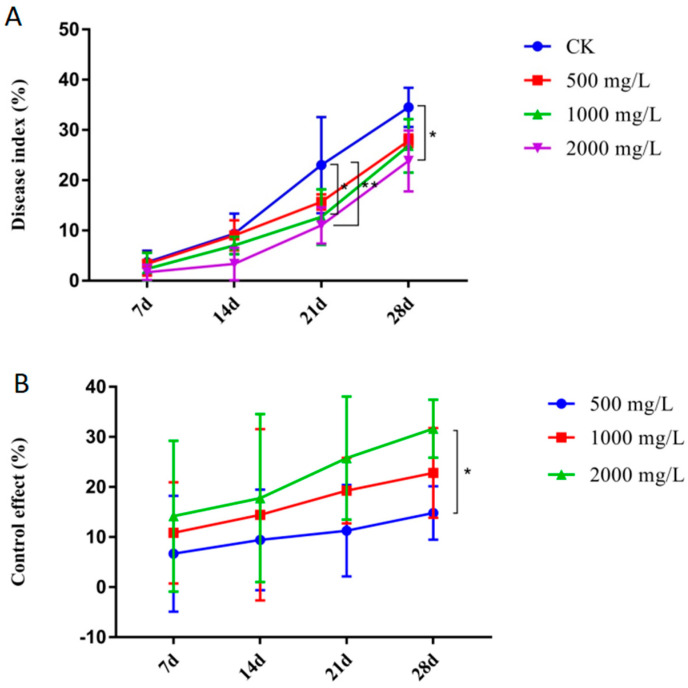
Disease index values (**A**) and control effects (**B**) in sugarcane with leaf scald disease at 7, 14, 21, and 28 d after inoculation with *Xa*. Plants were treated with one of two different Cu(OH)_2_ concentrations. Data are presented as the mean ± standard error (*n* = 3). * indicated significance with *p* ≤ 0.05 (ANOVA and Tukey’s HSD) and ** indicated significance with *p* ≤ 0.01 (ANOVA and Tukey’s HSD).

Firstly, we want to test the minimum amount of Cu(OH)_2_ used to effectively control sugarcane leaf scald disease. Sugarcane plants treated with 1000 and 2000 mg/L Cu(OH)_2_ during the same time period showed lower disease index values (26.8% and 23.8%, respectively) compared with the control group (CK, 34.5%) at 28 d after *Xa* inoculation. (Figure 1A). All three Cu(OH)_2_ treatment groups showed the highest efficacy at 28 d, with the 2000 mg/L treatment group showing 113.5% and 38.7% higher efficacy than the 500 mg/L and 1000 mg/L treatment groups, respectively (Figure 1B). The control effect of the 2000 mg/L Cu(OH)_2_ treatment did not show significant improvement compared with the 1000 mg/L Cu(OH)_2_ treatment. Thus, 1000 mg/L Cu(OH)_2_ was selected as the optimal bactericide concentration for subsequent experiments.

### 2.2. The Optimal Copper Hydroxide Concentration with Silicon Additive

**Figure 2:**
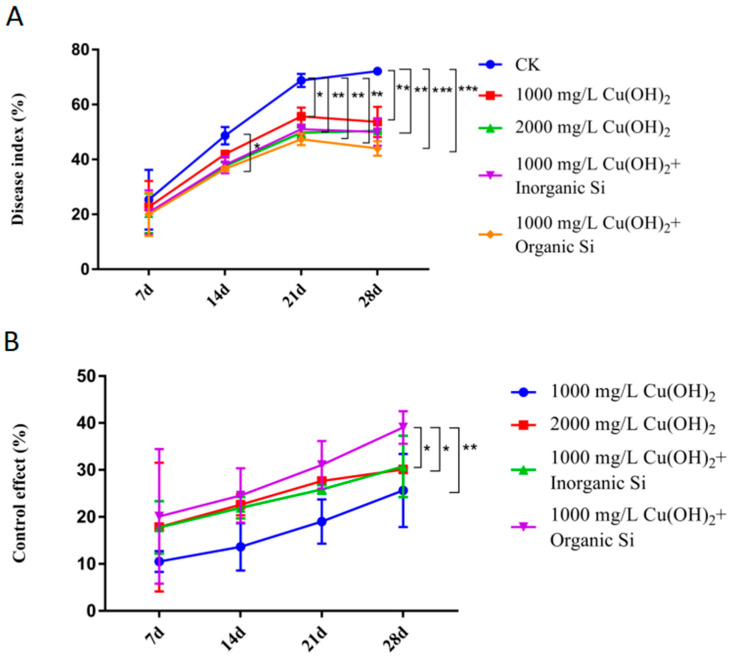
Disease index values (**A**) and control effects (**B**) in sugarcane with leaf scald disease at 7, 14, 21, and 28 d after inoculation with *Xa*. Plants were treated with one of two Cu(OH)_2_ concentrations and 0.05% inorganic or organic silicon. Data are presented as the mean ± standard error (*n* = 3). * indicated significance with *p* ≤ 0.05 (ANOVA and Tukey’s HSD), ** indicated significance with *p* ≤ 0.01 (ANOVA and Tukey’s HSD) and *** indicated significance with *p* ≤ 0.005 (ANOVA and Tukey’s HSD).

Five groups of treatments were set in this experiment: sugarcane treated with 1000 mg/L Cu(OH)_2_, 1000 mg/L Cu(OH)_2_ + 0.05% inorganic silica, 1000 mg/L Cu(OH)_2_ + 0.05% organosilicon silica, 2000 mg/L Cu(OH)_2,_ and control. All four treatment groups showed the highest control efficacy at 28 d. The control plants showed maximum disease index (72.2%) at 28 d after *Xa* inoculation, while sugarcane plants treated with 1000 mg/L Cu(OH)_2_ + 0.05% organosilicon during the same time period showed the lowest disease index (44.0%) compared with the control group. The group treated with 2000 mg/L Cu(OH)_2_ had a disease index of 50.4%, which was higher than the disease index of the group treated with 1000 mg/L Cu(OH)_2_ + 0.05% organosilicon; the disease index was reduced compared with the groups treated with 1000 mg/L Cu(OH)_2_ (by 22%), 1000 mg/L Cu(OH)_2_ + 0.05% inorganic silica (by 13.6%), and 2000 mg/L Cu(OH)_2_ (by 14.6%) (Figure 2A). The group treated with 1000 mg/L Cu(OH)_2_ + 0.05% organosilicon showed significant differences compared with the groups treated with 1000 mg/L Cu(OH)_2_, 1000 mg/L Cu(OH)_2_ + 0.05% inorganic silica, and 2000 mg/L Cu(OH)_2_; the control efficacy in the 1000 mg/L Cu(OH)_2_ + 0.05% organosilicon group was higher by 52.3%, 27.0%, and 43.1%, respectively (Figure 2B). Our data indicated the control effect of 1000 mg/L Cu(OH)_2_ + 0.05% organosilicon was even higher than 2000 mg/L Cu(OH)_2_; therefore, this treatment was selected for subsequent experiments.

### 2.3. The Optimal Timepoint for Copper Hydroxide and Silicon Additive Treatment

**Figure 3:**
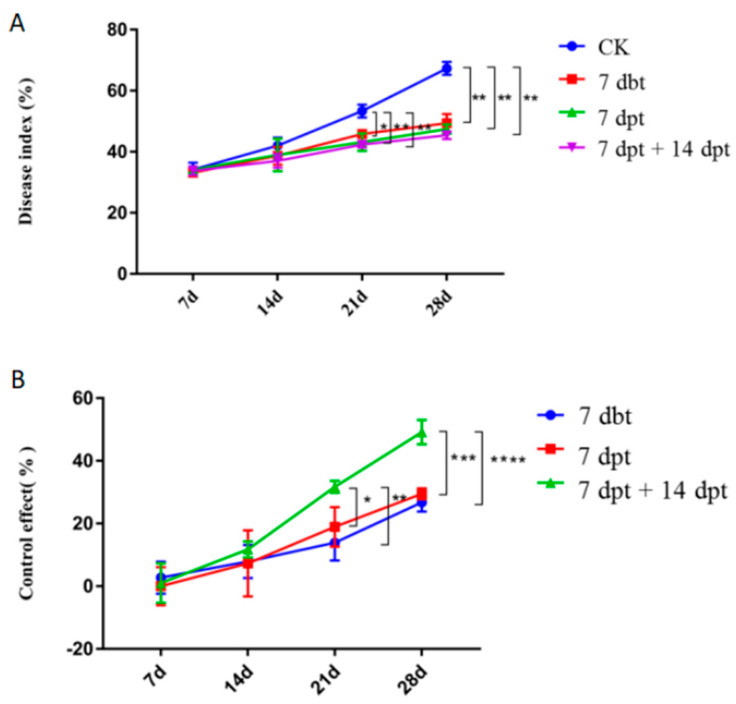
Disease index values (**A**) and control effects (**B**) in sugarcane with leaf scald disease at 7, 14, 21, and 28 d after *Xa* inoculation. Plants were treated with a synergistic bactericide at different time points before or after inoculation with *Xa*. dbt, days before treatment; dpt, days post-treatment. Data are presented as the mean ± standard error (*n* = 3). * indicated significance with *p* ≤ 0.05 (ANOVA and Tukey’s HSD), ** indicated significance with *p* ≤ 0.01 (ANOVA and Tukey’s HSD), *** indicated significance with *p* ≤ 0.005 (ANOVA and Tukey’s HSD) and **** indicated significance with *p* ≤ 0.001 (ANOVA and Tukey’s HSD).

The synergistic bactericide spray treatment was applied at 7 d before (7 dbt), 7 d after (7 dpt), or 7 d and 14 d after (7 dpt + 14 dpt) *Xa* inoculation. Control plants showed the maximum disease index (67.3%) at 28 d after *Xa* inoculation. Sugarcane plants in the 7 dpt + 14 dpt treatment group had the lowest disease index (45.4%) compared with the control treatment group for the same period (Figure 3A). All three treatment groups showed the highest control efficacy at 28 d, with 26.8%, 29.5%, and 49.2% improvements in control efficacy, respectively. The 7 dpt + 14 dpt treatment group showed the best control effect at 28 d, with significantly higher control efficacy values than the 7 dbt and 7 dpt treatment groups (by 83.6% and 66.8%, respectively) (Figure 3B).

**Figure 4:**
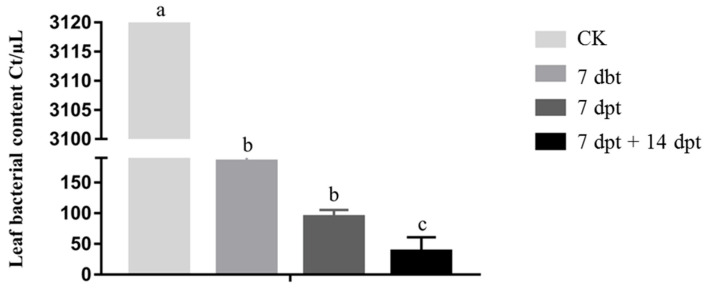
Bacterial content in *Xa*-infected sugarcane leaves at 28 d after inoculation. Plants were treated with synergistic bactericide at different time points before or after inoculation. dbt, days before treatment; dpt, days post-treatment. Bars represent the mean values; error bars indicate standard error (*n* = 3). Lowercase letters above the bars indicate statistically significant differences between treatments (*p* ≤ 0.05, ANOVA, and Tukey’s HSD).

Bacterial content was quantified in plants at 28 d after *Xa* inoculation. In control plants, the value was 3161.2 Ct/μL, which was 17.1, 33.6, and 83.9 times higher than in the 7 dbt, 7 dpt, and 7 dpt + 14 dpt treatment groups, respectively (Figure 4). The control group showed significant differences in bacterial content compared with all three treatment groups, with the 7 dpt + 14 dpt treatment showing the most significant inhibitory effect on bacterial content. Based on the results of the experiments described above, the optimal treatment protocol was determined: 1000 mg/L Cu(OH)_2_ mixed with 0.05% organosilicon, sprayed once at 7 d and once at 14 d after inoculation with *Xa*. These parameters were used in subsequent validation experiments in the field.

### 2.4. Verification of the Inhibitory Effects of the Optimized Bactericide Treatment Protocol on Sugarcane Leaf Scald Disease in the Field

**Figure 5:**
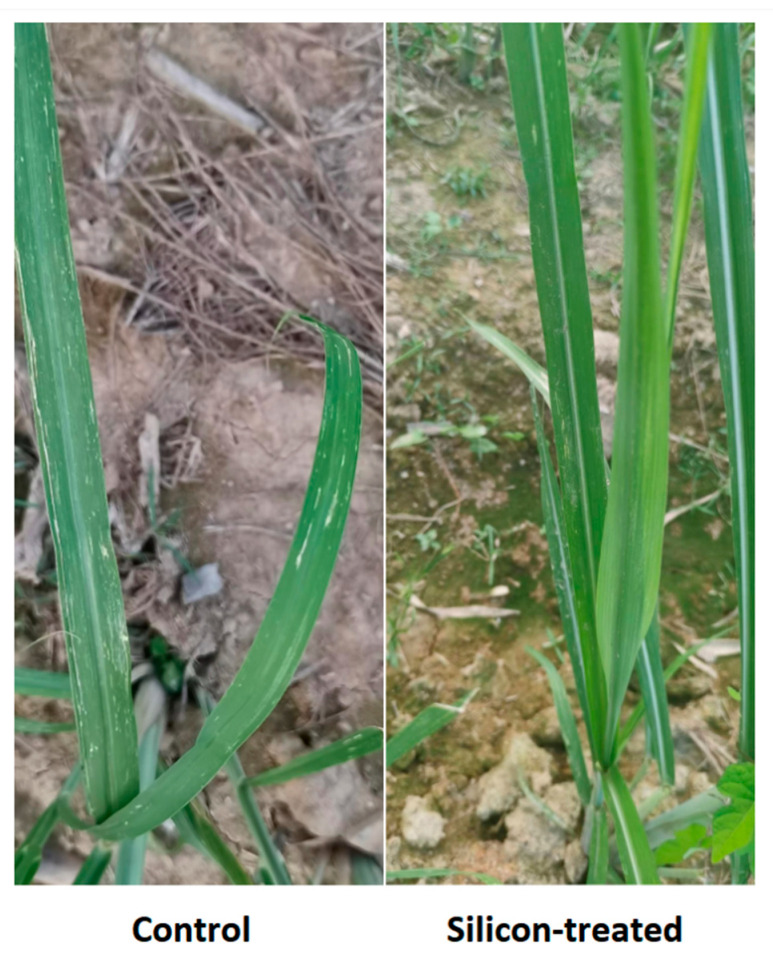
Symptoms of leaf scald disease on sugarcane against *Xa* infection in silicon-treated and control sugarcane plants.

**Figure 6:**
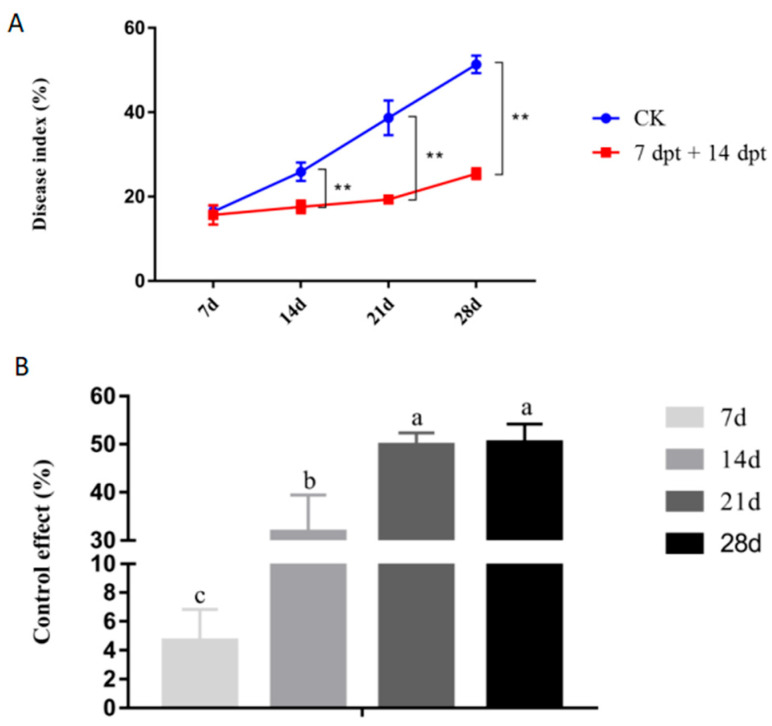
Disease index values (**A**) and bactericide control effects (**B**) in sugarcane with leaf scald disease. After treatment with synergistic bactericide at 7 and 14 d after inoculation with *Xa*. dpt, days post-treatment. Data are presented as the mean ± standard error (*n* = 3). Lowercase letters above bars indicate statistically significant differences between treatments (*p* ≤ 0.05, ANOVA, and Tukey’s HSD) and ** indicated significance with *p* ≤ 0.01 (ANOVA and Tukey’s HSD).

Sugarcane plants planted for 28 days in the field were inoculated with *Xa*, then treated with synergistic bactericide (1000 mg/L Cu(OH)_2_ + 0.05% silicon) at 7 and 14 d after inoculation. Three independent experiments were repeated in the year 2021. At 28 d after inoculation, the sugarcane leaf scald disease index was significantly higher in the control group than the treatment group (Figure 5); the disease index peaked at 51.3% in the control group at 28 d, which was 102.0% higher than in the treatment group (Figure 6A). The 7 dpt + 14 dpt treatment improved control of sugarcane leaf scald disease at 14, 21, and 28 d. The control efficacy stabilized by days 21 and 28, at which points the efficacy was significantly higher than it was at day 14. The control efficacy reached 50.3% at day 28, which was 9.8 times higher than it was at day 7 (Figure 6B).

**Figure 7:**
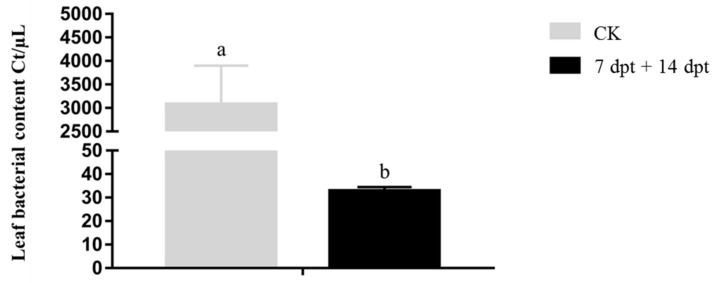
Quantitative measurement of bacterial content in leaves of *Xa*-infected sugarcane after treatment with synergistic bactericide at 7 and 14 d after inoculation. dpt, days post-treatment. Data are presented as the mean ± standard error (*n* = 3). Lowercase letters above the bars indicate statistically significant differences between treatments (*p* ≤ 0.05, ANOVA, and Tukey’s HSD).

Bacterial content was quantified in plants after disease index values were calculated. The control plants measured 3079.9 Ct/μL at 28 d after *Xa* inoculation, which was 93.6 times higher than in the 7 dpt + 14 dpt treatment group (Figure 7). In summary, bacterial content was significantly different in the control group than in the treated groups. Furthermore, the reduction in bacterial content was more significant in the field experiment compared with the growth chamber experiments.

## 3. Discussion

The results of the field experimental show that exogenous application of silicon with bactericide had a positive effect in controlling leaf scald disease in sugarcane. Although both Cu(OH)_2_ treatments significantly reduced the disease index, lower values were observed in plants that received a mixture of silicon and copper hydroxide. The results of the current study are consistent with those of Bathoova et al., who found that exogenous application of silicon with bactericide reduced the negative effects of Streptomyces in the sorghum rhizosphere [13]. The results of the present study are also supported by Mburu et al., who found that exogenous application of silicon with bactericide had similar effects in improving *Xanthomonas* wilt in banana [14].

Inorganic and organic silicons are usually studied as two different materials. The former is an absorbable source of the plant trace element silicon that directly induces various immune responses in plants. Previous results from our lab confirmed that exogenous application of inorganic silicons with bactericide can improve sugarcane resistance to leaf scald disease [15]. The organic silicons usually acted as an additive and mix with traditional bactericide. Inclusion of organic silicons in a bactericide mixture increases bactericide retention time on the crop surface, increases the amount of pharmaceutical retention, reduces the surface tension of liquid bactericides, and improves the resistance of an applied bactericide to rain washing. Together, these effects strengthen bactericide efficacy [16]. Here, we found that the combination of 1000 mg/L Cu(OH)_2_ and 0.05% organosilicon significantly improved the fungicidal effects of Cu(OH)_2_; it effectively reduced the occurrence of leaf scald disease and was more effective than application of 2000 mg/L Cu(OH)_2_ (Figure 2A,B). Similar results were obtained by Liu et al., who used Pyraclostrobin and Carmazine as fungicides to control Glomerella leaf spot. Using half of the optimal concentration of each fungicide led to significantly lower efficacy, but the efficacy returned to original levels when organosilicon additives were used. Furthermore, when the full concentration of each fungicide was used, the inclusion of organosilicon additives again resulted in significantly higher efficacy [17]. Zhang et al. studied the efficacy of organosilicon additives combined with 10 fungicides against egg sheath rust. For all 10 fungicides, the inclusion of an organosilicon additive reduced surface tension in the fungicidal agent, increasing adhesion, expanding the plant exposure diameter, and improving plant retention of the agent. These effects significantly reduced the amount of fungicide required while improving efficacy [18]. In potato, it was also found that reduced doses of kresoxim suspension had higher efficacy in treating late blight when silicon additives were used. Potato yield was not significantly different for those treated with the lower dose of kresoxim with silicon additives, and in some cases, yield was even higher than in those treated only with the conventional dose of kresoxim. Agricultural pollution was also significantly reduced in the treatment group that included silicon additives [19]. It has also been reported that both inorganic and organic silicon application can induce rice to acquire systemic resistance and enhance resistance to *Chilo suppressalis* and *Cnaphalocrocis medinalis* (Guenée) and result in significantly higher rice yield compared with untreated plants [20]. However, there is currently insufficient evidence that organosilicons can activate plant resistance, and additional experiments are therefore required in the future to further investigate these results.

The field production of crops typically requires consideration of the stages at which fungicides should be sprayed to control diseases. The results of this experiment show that the preventive effect conferred by spraying the optimal formula on sugarcane at an early stage was not significantly different compared with spraying at 7 d after inoculation with a disease-causing agent. However, the best control effect was achieved with one spray of the optimal formula at one week after disease onset and another spray at two weeks. Li Aiguo et al. applied silicon additives and different fungicide blends to wheat infected with sheath blight at three time points. The control effect of the combined treatment was improved after two or three applications compared with either fungicide application without silicon additives or a single application of the combined treatment; importantly, the amount of fungicide used in the combined treatment was 25% less than the amount used in the fungicide-only treatment [21]. Li et al. studied powdery mildew in moonflower using fungicides with and without a silicon additive. A fungicide was applied to plants in the field before disease onset, then once or twice after disease onset. The fungicide with the silicon additive was more effective by up to 23.1 percentage points compared with the other treatment groups, and water use was significantly reduced (by up to 50%). In addition to saving costs and time, reductions in water use prevent artificial increases in greenhouse humidity levels during the high-humidity season; this reduces free water residues on plant surfaces, preventing the spread of other diseases [22]. These reports are consistent with our findings in sugarcane experiments in the field; we found that the disease index was significantly lower in the group treated with fungicide and organosilicon than in the control group at day 28 (by about 26 percentage points), and the control effect was >50% in the treatment group (Figure 6 and Figure 7).

It was recently reported that bactericide activity can be influenced by changing the nature of the aliphatic substituent on the silyl group of the bactericide and its position in the phenyl group. Wei et al. generated a “homemade” synthetic compound, a fluorophenylether methylsilane derivative, which was compared with bactericides such as pyrrolofen and benzovidifluoropyr for inhibition of soybean rust. The “homemade” compound had C-2 in the distal benzene ring due to the trimethylsilyl component, which made it more effective in inhibiting pathogenic bacterial activity [23]. Some researchers have used a sol–gel/ultrasonic process to prepare MnSi-1 nanocomposites, which have a high relative surface area, to promote adsorption of bactericides to control bacteria and fungi and accelerate the antibacterial and antifungal responses; this process significantly improves bacterial inhibition compared with SiO_2_ treatment alone [24]. These reports show that silicon can significantly improve the suppressive effects of bactericides. Long-term use of the same bactericide commonly leads to resistant pathogens and reduced efficacy. Future research should therefore focus on the production of safer and more efficient silicon nanocomposites. Such compounds can increase the control effects of bactericides while reducing the negative ecological effects of pharmaceutical pollution in the field, ensuring safe, long-term control of sugarcane leaf scald disease in field production.

Previous studies have shown that copper hydroxide can effectively alleviate the disease index in sugarcane infected with *Xa* [7,8], but the excessive use of chemical agents can lead to bacterial resistance and environmental pollution. Consistent with the “3R theory”, here, we demonstrated a method to reduce the use of copper hydroxide at the source, using silicon as an adjuvant to suppress the development of leaf scald disease and improve plant resistance. Our study also showed that exogenous application of silicon with bactericide can improve the disease resistance of sugarcane while reducing the incidence of leaf scald, the disease index, and bacterial content in leaves. Silica additives are therefore promising candidates for environmentally responsible plant disease control via mixing with disease-controlling chemicals.

## 4. Materials and Methods

### 4.1. Materials

The *Xa*-susceptible sugarcane variety GT-58 was purchased from Kemik Agricultural Technology Service Company, Guangxi Province, China. Single-shoot groups were prepared from sugarcane stems, rinsed three times with distilled water, then soaked in distilled water at 25 °C for 24 h. The shoot groups were dried at room temperature for ~2 h before sowing. Experiments were conducted at the national engineering research center of sugarcane, Fujian Agriculture and forestry university, Fuzhou, Fujian, China. Plastic pots 55 × 28 × 10 cm in size (L × W × H) containing sugarcane stems were filled with 16 kg of peat soil (PINDSTRUP, Denmark) per pot. The peat soil had the following characteristics, as stated by the manufacturer: pH 5.5, 33 g/m^3^ ammonium nitrate, 91 g/m^3^ phosphorus (P_2_O_5_), and 158 g/m^3^ potassium (K_2_O). Seedlings were grown under a 16/8 h light/dark photoperiod in a climatic chamber at 28 °C with 60% humidity. Experiments were conducted in a completely randomized design with three replications; each pot (containing 32 plants) was considered one replicate. No additional fertilizer or other micronutrients were applied during the growth period (about 30 days after sugarcane sprouting). The inorganic silicon type was K_2_SiO_3,_ organosilicon (ethoxy-modified trisiloxane) was purchased from Muqi Trading Co., Ltd. in Shandong Province, China, and Cu(OH)_2_ was purchased from Zhongke Xinchuang Biotechnology Co., in Fuzhou, Fujian Province, China.

### 4.2. Preparation of Xa, Plant Inoculation, and Leaf Sampling

Single colonies of the *Xa* strain FJ1 were suspended in 1 mL of *XAL* (*Xanthomonas albilineans* liquid medium) and incubated at 28 °C for 48 h with shaking at 200 rpm [25]. Aliquots of culture (1 µL each) were added to 40 mL fresh *XAL* medium and incubated at 28 °C for ~10 h. The bacterial suspension was diluted to 10^8^ CFU/mL, and the tips of sugarcane leaves were removed using the leaf-cutting method after dipping scissors in the *Xa* suspension. Sugarcane seedlings at the three- to five-leaf stage were inoculated or sprayed with distilled water as a control [6]. Leaves were harvested at random from *Xa*-infected sugarcane seedlings at the time points appropriate for each experiment. The +1 leaves were collected, frozen in liquid nitrogen, and stored at −80 °C.

### 4.3. Screening of Optimal Formulas for Bactericide Application in Laboratory

We first screened suitable concentrations of the bactericide Cu(OH)_2_ for disease control. Cu(OH)_2_ (diluted to 500, 1000, or 2000 mg/L with water) was sprayed to plants at 7 d after *Xa* inoculation. Disease index values were recorded for each group at 7, 14, 21, and 28 d after inoculation. These data were analyzed to determine the optimal inhibitory concentration of Cu(OH)_2_. There were three biological replicates for each group. Control plants in this experiment were treated with the same amount water.

The effects of silicon additives were next tested using the optimal inhibitory concentration of Cu(OH)_2_. A previous study showed that 0.05% organosilicon combined with a fungicide was effective in controlling Magnaporthe grisea [26]. We therefore tested the effects of Cu(OH)_2_ added with 0.05% silicon to determine synergistic effects. Solutions were prepared for five treatment groups: control (CK, water); 1000 mg/L Cu(OH)_2_; 2000 mg/L Cu(OH)_2_; 1000 mg/L Cu(OH)_2_ + inorganic silicon; and 1000 mg/L Cu(OH)_2_ + organic silicon. Disease index values were recorded as described above. There were three biological replicates for each group.

Finally, we evaluated the optimal timing for application of the optimal formula. This was conducted with a control group and three treatment groups: plants sprayed with the optimal formula one week before inoculation, plants sprayed one week after inoculation, or plants sprayed both one week and two weeks after inoculation.

### 4.4. Verification of Bactericide Inhibitory Effects on Sugarcane Leaf Scald Disease in Field

The trial site for the field experiment was located at the sugarcane experimental base of Fujian Agriculture and Forestry University. There were six plots, each 30 m long and 1 m wide. Each plot contained 30 sugarcane plants per plot, which were divided into three treatment groups and three control groups. The bactericide formula used was 1000 mg/L Cu(OH)_2_ + 0.05% organic silicon. Bactericide was sprayed on sugarcane plants at 7 and 14 d after inoculation with *Xa* such that the leaves were well-moistened. Plants in the control group were treated with water. Disease index values were recorded at 7, 14, 21, and 28 d after inoculation. Sugarcane leaves were randomly sampled at 28 d after inoculation and stored at −80 °C for absolute quantification of *Xa*. Three replications were performed in the base; each independent experiment was 1 m away from each plot. The data were analyzed to verify the inhibitory effect of the optimal formula on sugarcane leaf scald disease under field conditions.

### 4.5. Disease Index Recording and Data Analysis

Leaf scald disease grades were observed and recorded for control and treated sugarcane seedlings at 0, 7, 14, 21, and 28 d after *Xa* inoculation. Disease grades were assigned as follows: no symptoms (grade 0), one or two white pencil marks (grade 1), more than two white pencil marks (grade 2), foliage greening or yellowing (grade 3), leaf necrosis (grade 4), or plant death (grade 5) [27]. The disease index was calculated as follows:

∑ (number of diseased plants of each grade × representative value of each grade)/(total number of plants surveyed × representative value of highest grade)

The percentage of disease incidence was calculated as:

(number of diseased plants/total number of plants surveyed) × 100

The percent control effect was calculated as:

[(disease control index − silicon processing disease index)/disease control index] × 100

The mean values between treatment groups were assessed for statistically significant differences using analysis of variance (ANOVA) with the statistical tool “GraphPad Prism7” (HuanZhongRuiChi Technology Co., Ltd., Beijing, China)and post hoc Tukey’s honestly significant difference (HSD) test. Differences between groups were considered significant at *p* ≤ 0.05.

### 4.6. Quantitative Analysis of Bacterial Content

DNA was extracted from leaves using the CTAB method. Bacterial content at 28 d after *Xa* infection was determined by quantitative reverse transcription (qRT)-PCR using the TaqMan probe 5′(FAM)-TGCTCGCGAGAGCGCTCTCTACA-(Tamra)-3′ and gene-specific primers (F: 5′-GCGATCTCGTTGTTGATGCG-3′, R: 5′-CGGCCAGAAGCAGAATCC-3′) as described by Shi et al. [28]. The qRT-PCR assay was performed in a 20 µL reaction mixture; each reaction contained 1 µL DNA or cell suspension, 10 µL FastStart Universal Probe Master (Roche Diagnostics Ltd., Shanghai, China), 0.4 µL of each primer (XaABCR3 and XaABCR3) (10 pmol/L), 0.8 µL of TaqMan probe (XaABCP3) (10 pmol/L), 0.2 µL of ROX Reference Dye II (50×), and 7.2 µL ddH_2_O. The PCR program consisted of an initial denaturation of 2 min at 95 °C, 40 cycles of 5 s at 95 °C, and annealing and extension for 30 s at 60 °C. When the cycle threshold (Ct) value was <35, the test result was considered positive. When the Ct value was ≥ 35, the test result was considered negative. Bacterial content was calculated using the Standard Curve method with the following formulas:

z (copies/µL) = 10y

y = −3.2384x + 38.854

where z is the bacterial content and x is the Ct value.

## 5. Conclusions

In this study, we discovered that 1000 mg/L Cu(OH)_2_ mixed with 0.05% organosilicon additive applied twice (7 days after disease appearance) successfully reduced the sugarcane leaf scald disease index. Furthermore, this selected method was also verified in the field experiment. Our results demonstrate the addition of organosilicon in the chemical bactericide reduced pathogenic bacteria content and decreased the disease index, even at a lower concentration. Therefore, we provided a practical example of environmentally friendly chemical control for sugarcane leaf scald disease.

## Data Availability

Not applicable.
